# A Case of Injury to the Median Nerve

**Published:** 1868-08

**Authors:** J. R. Lothrop


ART. II.—*A Case of Injury to the Median Nerve.* By J. R. Lothrop, M. D.

This occurred in a young girl about 10 years of age. When the case first came to my notice, it seemed to resemble in many respects a rheumatic affection of the hand. There was a good deal of swelling and tenderness on the back of the hand and about the wrist. There was, however, no observable redness.

Subsequent and more careful examinations led to the conclusion that it belonged to the class of nervous affections. The hand was swollen and tender, but when closely examined it was found that the tenderness did not affect the whole hand. Moreover it was not only limited, but at some points it was so extreme as to give rise to pain upon the slightest touch. The fingers, all of them, were drawn towards the palm, as in closing the hand, but not in contact with it. The thumb was slightly flexed, but not in contact with the forefinger. The slightest touch upon the ends of the first three fingers was intensely painful, the patient saying that it felt like fire. The little finger, however, could be touched and extended even, without pain, but it became flexed as soon as released. The thumb partook of the exalted sensibility of the first three fingers. The backs of the thumb and three fingers, especially about the nails, and portions of the surface of the palm were sensitive to the touch. Pressure upon the anterior surface of the forearm excited pain, which was felt, rather deeply, as far up as the bend of the elbow, at which point it caused increased pain and shooting pains up and down the arm, but particularly downwards. At this point also there soon appeared swelling, hardness, and discoloration, marking it as the point of injury. Pressure above the elbow was not attended with any notable painful sensation. The exalted sensibility of the ends of the thumb and first three fingers, or, as it may be styled, hyperæsthesia, was peculiar and interesting. The tactile sensibility, always normally delicate, was so much exalted as to become painful, and the lightest touch was even more so than when the parts were seized rather roughly. For instance, if any body was lightly brought in contact with them, the hand was quickly drawn away as if the body were hot, and in fact the sensation was as of heat. Any attempt to extend the fingers caused intense suffering. When the hand was at rest there was constant pain, varying in intensity, which was felt in the arm, hand, and fingers, and by depriving the patient of sleep, began to have a perceptible influence upon her health. After a time, however, a number of weeks, the violence of the symptoms was over, the fingers could be handled without causing pain in any great degree, and it was not long before they could be fully extended and remain so. In time the hand entirely recovered its normal sensation and motion.

As to the cause of the injury there was some obscurity. The patient could only remember that, a short time before the arm began to be painful, she had been pulled quite violently by the hand; and that at the time she felt some temporary pain. The violent tension of the surrounding parts and perhaps of the nerve itself at the bend of the elbow, probably were the cause of the subsequent phenomena. Certainly the swelling, tenderness, and discoloration at that point were indicative of some trouble there. The inflammation of the surrounding tissues may have involved the nerve, or the symptoms may have been due in some measure to pressure.

In the treatment of such a case little can be done beyond allaying the pain till recovery takes place; and the tendency is to recovery, in time, longer or shorter, according to the nature of the injury. Cold is always a relief to the sense of heat which is usually felt. Anodyne lotions sometimes soothe for a short time, absolute rest of the limb and protection by wrappings, covered on the outside by oiled silk. Opium is indispensable, and the best method of using it, is by subcutaneous injection. If the affection is one liable to recover in a short time, these measures are the best, but if it continues, division or excision of the nerve is, by all means, to be advised. Even if union of a divided nerve is attended with a recurrence of the pain, still there is something gained by an interval of freedom from it. In the case above related, cold water, anodyne liniments, containing tincture of aconite and of opium, were employed externally, and opium internally. They had probably very little agency in hastening recovery, their benefits being limited to the production of partial relief. They may in some slight degree have controled the inflammation at the bend of the elbow, and hence been of benefit.

This case was clearly one of injury to the median nerve, at the bend of the elbow. That the median nerve was injured, was clear from the parts affected. That the injury was at the bend of the elbow was evident from the local signs there, and from the fact that the muscles of the forearm were affected. This last circumstance might have resulted from an injury higher up, but in connection with the local signs, it was indicative of injury at that point. The injury was only severe enough to exalt, but not destroy the function of the nerve. Hence, the nerve having a double function, viz.: of sensation and motion, there was observed increased sensibility in the parts which depend upon it for sensation, and contraction of those muscles to which its motor fibres are distributed. The sensory fibres of the median are distributed to the fore, middle, and ring fingers, and the sentient extremity of the thumb, so that those parts derive their tactile sensibility from it, while the little finger gets its sense of touch from the ulnar nerve. This peculiarity of distribution explains the observed fact, that the little finger could be touched without exciting painful sensation, while the other fingers were affected with extreme hyperæsthesia. On the other hand the little finger was equally with the others drawn into the palm, in a state of constant flexion, a condition explained by the fact that the motor fibres of the median nerve are distributed to the flexors of the forearm, which govern the flexion of all the fingers.

The diagnosis of the seat of injury and the particular nerve affected, was made upon the phenomena above stated. The median nerve is a source of motor influence to all the fingers and the thumb, while it affords sensory influence to the first three only, together with the thumb. In any case in which observed facts are in accordance with the above statement, we seem to have a rational ground for attributing the injury to the median nerve. As a matter of practical interest, it is probably of no great importance that there should be such nicety in the diagnosis. Very few cases would be likely to occur to any practitioner in which it would be possible to make out clearly the particular nerve injured. Injuries to the hand and forearm are seldom so limited. But they sometimes occur, especially in gun-shot wounds. Even then their chief interest lies in the aid which clinical facts can bring to experimental physiology. With, however, pretty careful observation, it may not be possible to arrive at exact conclusions. Recent statements and observations as to the effects of division and exsection of nerves, do not seem to confirm some opinions which have been held in regard to sensation and motion, as dependent upon the distribution of particular nerves. Thus it has been stated that exsection of any one nerve, as for instance the median, has not destroyed the sensibility of those parts of the hand to which it is distributed, and on the other hand after exsection, sensibility, when destroyed, has been entirely restored long before there has been a regeneration of the lost portion of the nerve. If these statements are correct, the teachings in regard to sensation, founded upon the distribution of the nerves, are entirely wrong. There is reason to believe, however, that in many of these cases the statements are not based upon accurate and careful observations. In cases of injury of any one nerve, either the radial, ulnar, or median, the pain is not always located with exactness by the patient, but is in a general way referred to the hand. A very careful observation on the part of the surgeon, might and probably would localize it, to correspond with the nervous distribution. What is here stated of exalted sensibility or pain following injury, would probably be found to apply to loss of sensibility or numbness following division or excision.

There are numerous cases reported in which loss of sensation or motion is never fully restored after division or excision of a nerve, the liability of failure being greater after the latter, or in fact of the loss remaining permanent. This liability to incomplete repair, if not entire failure of it, is a matter always to be considered by the surgeon, when circumstances seem to call for the operation of excision. The actual removal of any considerable portion of the substance of a nerve, as a remedial measure, is always undertaken with the expectation that the restoration of the nervous function, entire or incomplete as may be, if it take place at all, can only take place after a long time. This is the ordinary experience of surgeons, yet M. Paulet, in a memoir read before the Paris Surgical Society, recently stated as the result of his examination of recorded clinical experience, that he found in some cases of excision, the functions were very soon re-established, and in some, excision of an important nerve in nowise disturbed sensation or voluntary motion. We are then compelled to distrust the accuracy of the recorded facts derived from clinical experience, or physiologists are in the wrong and must study nervous phenomena in some other light than that of distribution. We are disposed to feel, with the late lamented Dr. Warren, that "the connection between the precise nature of the injury and the subsequent phenomena has not, often, been marked out with so much exactness as could be desired." Surgeons will not as yet be prepared to perform and recommend exsection of an important nerve with the expectation that no effect will be perceptible, as to motion or sensation, in the parts supplied by the nerve. The case related above has no bearing upon the question of excision, and therefore furnishes no proof either for or against the interruption of nervous function by the operation. But it shows that exaltation of the function of a nerve is in accordance with its distribution, from which we may infer that any decrease of function would be exhibited in the same direction.

Not long since a medical man related to me his personal experience of an affection of a nerve of the hand, very much like the case related above, which appeared to have been an injury of the median nerve, from muscular effort. A short time after, driving a spirited horse that required great mnscular effort of the arm to control, he began to experience pain in the hand and arm. The pain extended along the middle of the forearm, into the palm, where the pain was intense, burning in character; and also into the ends of the fingers, the little finger not being affected. There was flexion of the fingers and thumb. The pain was constant, and severe in paroxysms. The only relief was obtained by free use of morphia. As to external applications he found hot poultices most comforting. The tactile sensibility was so much exalted that contact with the poultices, especially when the fingers were first lightly laid upon them, caused burning and pain. The same feeling of heat was caused by contact with any body. In about two months the hand was restored to normal motion and sensation, except the middle finger, which still retained a tendency to flexion when not forcibly extended.

The following case, taken from Romberg, who quotes it from Denmark, though it is related as a case of injury to the radial nerve, appears to answer more nearly to a case of injury of the median: A healthy young soldier was wounded at the storming of Badjos, by a musket ball which entered the triceps one inch and a half above the inner condyle of the humerus, grazed the bone, passed downwards and outwards and made its exit in front near the bend of the elbow. The wound healed. Some time after, he began to suffer pain, which became so excessive that opiates would not relieve it, and sleep was almost entirely absent. The forearm was bent, the wrist also, and hand flexed. Pressure upon the cicatrix caused additional torture. He described the sensation of pain as beginning at the extremities of the thumb and all the fingers *except the little one* and extending up the arm to the point wounded. It was of a burning nature, he said, and so violent as to cause a continual perspiration from his face. His sufferings were so great that he requested the removal of the arm by amputation, which was performed with quick relief. The surgeon who reported the case wrote, "the part wounded, the nature of the pain, and its course from the fingers, with the *exception of the little*

*one*, indicated the affection to be in the radial nerve." From the facts stated in regard to the fingers it would seem that affection of the median, rather than the radial nerve, was indicated. The remedy employed, namely, amputation, would hardly be advised by a surgeon of modern times, at least not until he had made a trial of exsection. Amputation, however, in the case of smaller members, as portions of fingers and toes, is more worthy of notice.

I am permitted to refer to a case which came under the care of Dr. Hutchins, of this city, in which amputation of a portion of a finger was resorted to with the happiest results. A young lady met with an injury to the end of the little finger. In moving a table, her fingers being over the edge, the little finger was caught between the table and the wall of the room. Blood was affused under the nail and the injury was followed by severe pain shooting up the arm in the course of the ulnar nerve. This pain, with intervals of freedom, continued for many months, interfering seriously with the health. During its continuance a great variety of treatment had been *endured*, blistering over the spinal column among other measures. When first seen and examined by Dr. H., he found that the pain originated in the little finger and could be excited, at any time, by pressure upon the nail, over what appeared to be a small cyst under it. An attempt was first made to remove the trouble by removing the nail and cutting out the diseased tissue under it; but this procedure failing to relieve the pain, after a short time Dr. H. decided to amputate the finger at the last joint. The operation was performed and the relief afforded complete. The pain ceased and there was soon manifest a marked improvement in health. I assisted at the operation and examined the part removed, which consisted of the distal phalanx and the tissues upon it. There was found under the nail a small, dense, diseased substance, which had by pressure produced a small depression in the end of the bone by caries or absorption. This diseased mass had involved the delicate fibres of the sensitive nerve, and hence the pain.

Speaking of pressure upon a nerve as a means of exciting pain, Romberg says, "it can only be if, at the same time, there is irritation and traction of the fibres, consequently it is much more frequent in interstitial tumors, though their bulk be ever so small, as in painful tubercle, than in large neuromatous growths, over which the sensitive fibres may be spread fan-like, and be stretched to the utmost without marked derangement. Compression of a healthy nerve of sensation, by tumefaction of adjacent parts, interrupts conduction and induces anæsthesia. If the same nerve becomes inflamed or ulcerated, pressure causes severe pain, as we occasionally see in aneurisms."

If such is the case, we must eliminate pressure as a cause of the pain in the case related at the beginning of this article, and conclude that there must have been some irritation or inflammation of the nerve to excite such an extreme exaltation of sensation, and the increase of motor influence shown by the contraction of certain muscles of the hand and forearm.

Dr. Brown-Séquard groups the symptoms which are caused by lesion of a nerve in two classes. In one, the symptoms are the effects of the loss of function of the nerve; in the other, they are due to some action of the injured or irritated nerve. In one, the effects are cessation of action; in the other, increase of action. Hence, on the one hand, there is paralysis, and on the other, contraction of muscles, pains, prickings, altered sensations as of heat or cold, etc.

It is not in keeping with our present purpose to consider any effects but those which are caused by irritation or inflammation of nerves. These are of two kinds: direct and indirect or reflex. The direct are experienced along the nerve, or at its ramifications, in the course of its distribution. Often these are the only symptoms found. But the direct and reflex symptoms may exist at the same time; then, in addition to the contraction, pain, etc., referable to the particular nerve injured, those varied and wonderful nervous affections, such as epilepsy, tetanus, convulsive affections, neuralgia, etc., some of them truly grave and formidable, which are the effects of that powerful and inexplicable influence we call reflex, will be produced. The reflex symptoms are not always in proportion to the direct. Pain may be very intense as a direct effect, and yet no remote reflex symptoms be developed. This is common enough in our experience. The case related above of the soldier who was injured by gun-shot wound is one in point. In that case the pain was spoken of as agonizing, and yet no serious reflex actions were excited. On the other hand, grave reflex affections may arise, when the direct symptoms have almost escaped attention. In fact, serious reflex disturbances may be excited by an irritation of a nerve, of which there had been no sensation. From what has been stated it can be seen that irritation of a nerve, existing as a cause, may, in some cases, have no reflex effect whatever, and in others, excite the most varied and serious disturbances.

In treating the effects of irritation, Dr. Séquard insists, that the previous existence of a lesion of nerve, as a starting-point, must not be forgotten. Search should be made for it by pressure along the course of the nerve, as the only means of discovering it. Of the means to be employed, the best theoretically are the best in fact. First in benefit, is the division of the affected nerve; or exsection, if there is likely to be reünion before the irritation ceases, or if the nerve is altered. Success will follow this measure even when two inches of nerve are removed; but a number will fail. Second in benefit, is subcutaneous injection of narcotics, on or near the irritated nerve: morphine and atropia, separately or combined, may be used; half a grain of the former and a sixtieth of the latter. Amputation is hardly to be advised except in affections of the extremities of fingers or toes, which afford a starting-point for serious reflex actions. Then, as has been seen, it may be resorted to with great benefit, or complete relief. Dr. Séquard has no faith in the antagonism of opium and belladonna, and is very careful to caution against reliance upon such action in cases of opium poisoning.

